# The first competing risk survival nomogram in patients with papillary renal cell carcinoma

**DOI:** 10.1038/s41598-021-91217-z

**Published:** 2021-06-04

**Authors:** Xing Su, Niu-Niu Hou, Li-Jun Yang, Peng-Xiao Li, Xiao-Jian Yang, Guang-Dong Hou, Xue-Lin Gao, Shuai-Jun Ma, Fan Guo, Rui Zhang, Wu-He Zhang, Wei-Jun Qin, Fu-Li Wang

**Affiliations:** 1 Department of Urology, Xijing Hospital, Fourth Military Medical University, Xi’an, 710032 China; 2 Department of Thyroid, Breast and Vascular Surgery, Xijing Hospital, Fourth Military Medical University, Xi’an, 710032 China; 3 Department of Cardiology, Xijing Hospital, Fourth Military Medical University, Xi’an, 710032 China; 4 Department of Urology, The 986th Hospital of Air Force, Xi’an, 710054 China

**Keywords:** Oncology, Urology

## Abstract

There is still a lack of competing risk analysis of patients with papillary renal cell carcinoma (pRCC) following surgery. We performed the cumulative incidence function (CIF) to estimate the absolute risks of cancer-specific mortality (CSM) and other-cause mortality (OCM) of pRCC over time, and constructed a nomogram predicting the probability of 2-, 3- and 5-year CSM based on competing risk regression. A total of 5993 pRCC patients who underwent nephrectomy between 2010 and 2016 were identified from the Surveillance, Epidemiology, and End Results (SEER) database. The 2-, 3-, 5-year CSM rates were 3.2%, 4.4% and 6.5%, respectively, and that of OCM were 3.2%, 5.0% and 9.3%, respectively. The estimates of 5-year cumulative mortality were most pronounced among patients aged > 75 years in OCM (17.0%). On multivariable analyses, age, tumor grade, T stage, N stage, and with or without bone, liver and lung metastases were identified as independent predictors of CSM following surgery and were integrated to generate the nomogram. The nomogram achieved a satisfactory discrimination with the AUC_t_ of 0.730 at 5-year, and the calibration curves presented impressive agreements. Taken together, age-related OCM is a significant portion of all-cause mortality in elderly patients and our nomogram can be used for decision-making and patient counselling.

## Introduction

Renal cell carcinoma (RCC) is a common malignancy of genitourinary system, and accounts for approximately 2–3% of all malignancies in adults^1^. After clear cell RCC (ccRCC), papillary renal cell carcinoma (pRCC) is the second most common histological subtype of RCC, accounting for 6–18% of kidney tumors^2^. According to the clinical and biological distinction, pRCC could be subdivided into two types^3^. Type 1 is usually multifocal, characterized by basophilic cytoplasm, small and uniform nuclei while type 2 is heterogeneous, consisting of eosinophilic cytoplasm and large spherical nuclei^3,4^.

The postoperative outcome of RCC has been extensively studied. To date, several clinical prognostic models have been developed to predict the overall survival (OS), the cancer-specific survival (CSS) and the progression-free survival (PFS) after nephrectomy^5–7^. According to the Heisenberg classification system in 1997^8^, it is recognized that RCC is not a single entity of tumor, but rather consists of four major histological subtypes with distinct biological behavior, clinical course and oncologic outcomes^9–12^. However, most studies often focus on ccRCC only. With respect to pRCC, only a few tools have been developed to predict the survival outcomes by using different prognostic parameters and end-points over the last decade^13–15^, which remains controversial. Moreover, outcomes in survival research are frequently confounded by competing events that affects the interpretation of the primary event of interest^16^. For example, when the cancer-specific mortality (CSM) of pRCC is the primary end-point of interest, other-cause mortalities (OCM) as competing events and can affect the calculation of the overall survival benefit after treatment. Previous studies have demonstrated that the all-cause mortality of RCC continues to rise despite early diagnosis and aggressive intervention^17,18^. Given the higher 5-year CSS rates in pRCC patients compared to ccRCC^19^, age-related OCM could not be overlooked in this population, especially for elderly patients with comorbidities^17,20^, who may benefit little from invasive surgery and die from other diseases. In this era of emphasis on individualized therapy, it is critical to differentiate between risk factors for cancer and noncancer death to help better risk-stratify pRCC patients following surgery. Besides surgical intervention, clinicians should be aware of the underlying diseases and provide further supportive treatment. Specifically, traditional Kaplan–Meier and Cox methods may significantly overestimate the risk of cancer-specific death in the presence of competing events^16,21^. Thus, the competing risk method is more suitable for constructing a clinical prognostic model of survival data nowadays^16^. To the best of our knowledge, a competing risk prognostic nomogram specifically for pRCC has not yet been developed.

For the above reasons, the aims of the present study were to use the competing risk method to explore the independent predictors for cancer-specific death in pRCC patients based on the Surveillance, Epidemiology, and End Results (SEER) database and to develop a competing risk nomogram for clinical decision-making and patient counselling.

## Patients and methods

### Study population

Data of patients with pRCC (ICD-O-3 site code C64.9 and histology code 8260/3) between 2010 and 2016 were extracted from the SEER database (2004–2016 dataset), which contains the population-based cancer incidence information from 18 registries and covers nearly 28% of the United States population^22^. To ensure at least one year of follow-up, we excluded patients who were diagnosed after December 31, 2015. A total of 9690 patients were identified through the SEER*Stat software (username: 10646-Nov 2018).

**Figure 1:**
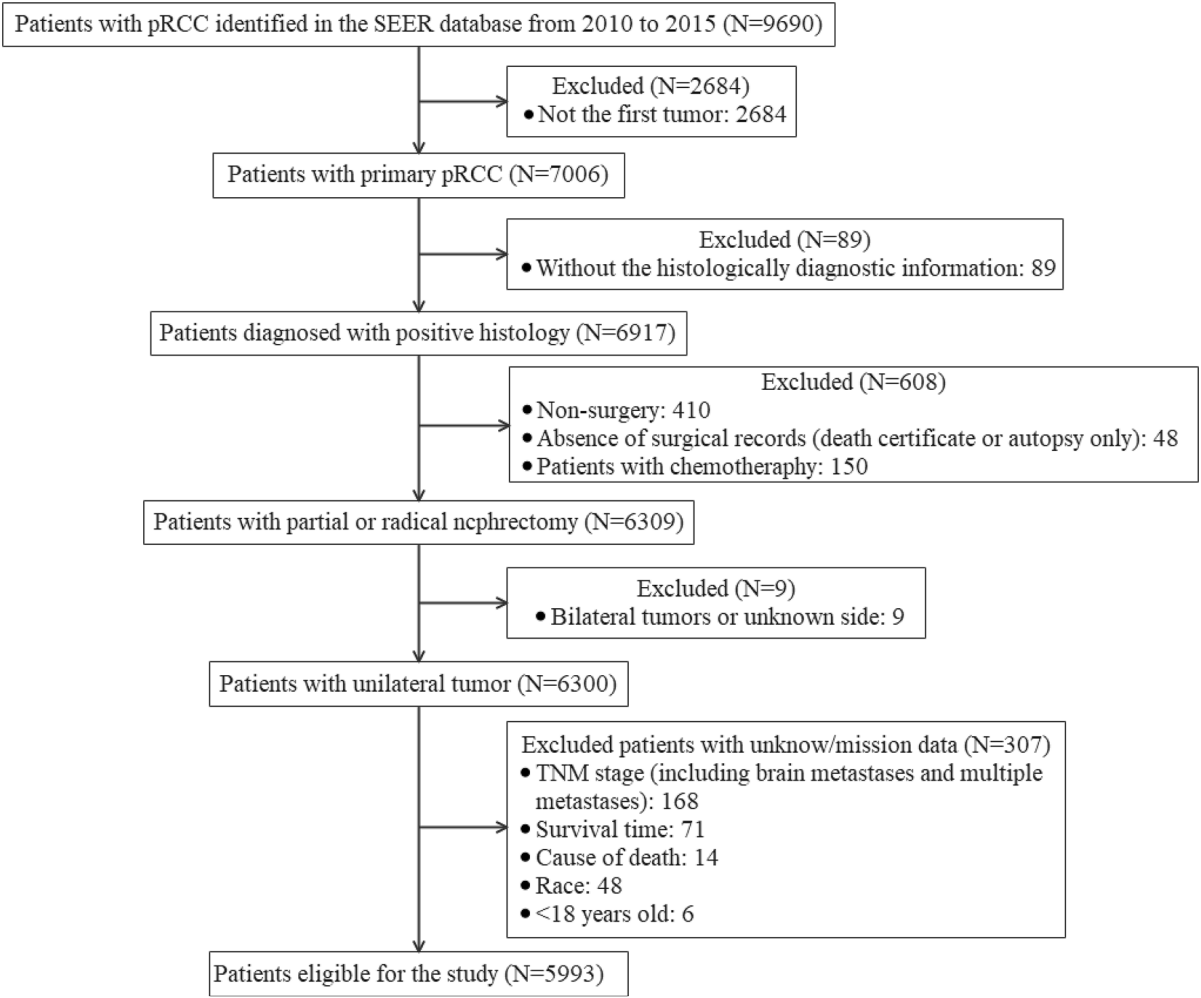
The flowchart of patient selection in the SEER database. *pRCC* papillary renal cell carcinoma.

The inclusion criteria of eligible patients were as follows: (1) primary pRCC; (2) diagnosis with positive histology; (3) underwent partial or radical nephrectomy. (4) Unilateral tumor. (5) Aged over 18 years. The exclusion criteria were patients who received chemotherapy, and with unknown or missing information on surgery records, tumor side, TNM stages, cause of death, race and survival time. In addition, we also excluded patients with brain metastases (N = 7) and multiple metastases (N = 7) as the cases were very few. Eventually, a total of 5993 patients were eligible in the present study. The detailed flowchart of patient selection is shown in Fig. 1.

Since the SEER database provides open and free access, no informed consent is needed and the current study adhered to the Declaration of Helsinki and its amendments.

### Covariates, end-points and follow‑up information

The demographic and clinicopathological variables in our study included sex, race, age at diagnosis, laterality, tumor grade, T and N stage, and M status (presence of liver, lung and bone metastases or not). Specifically, age was divided into the following groups: < 50 years (the young group), 50–74 years (the middle group) and > 75 years (the elderly group); race was classified into white, black, and others; histological grades were categorized into the following five groups: grade I (well differentiated), grade II (moderately differentiated), grade III (poorly differentiated), grade IV (undifferentiated) and unknown; and according to the 2010 American Joint Committee on Cancer (AJCC) classification, we classified T stage as T1, T2, T3 or T4 and N stage as N0 or N1, respectively. The end-points were CSM and OCM. The censored data were defined as patients who were still alive from the date of diagnosis to the date of last follow-up or December 31, 2015. The follow-up time for each patient was measured with the survival time observed in the dataset.

### Statistical analysis

Continuous variables were reported as medians and interquartile ranges, while categorical variables were summarized as frequencies and proportions percentages. In data analysis, OCM was considered as a competing event for CSM, and the effect of each variable on the absolute risk of different outcomes was estimated using the Fine and Gray’s competing risk model^23^. At first, we performed the cumulative incidence function (CIF) to describe the probability of each event among the categorical variables over time and plotted the corresponding CIF curves at the same time. The differences within the subgroups were assessed by Gray’s test^24^. Second, significant variables in univariable analysis (*P* < 0.05) were selected to fit the optimal proportional subdistribution hazard model using a backward elimination method and the 2-, 3-, and 5-year prognostic nomogram for CSM was further generated based on the significant model coefficients^25^. Finally, the predictive performance of our nomogram was internally validated via bootstrapping with 1000 resamples. The discrimination was measured by the time-dependent area under the receiver operating characteristic (ROC) curve (AUC_t_)^26^. Subsequently, the 2-, 3-, and 5-year calibration curves were plotted to visually compare the nomogram-predicted probabilities with the observed CSM rates.

We used SPSS 23.0 (IBM Corporation, Armonk, NY, USA) for descriptive statistics, and the statistical analyses mentioned above were performed using R software (version 4.0.3, R Core Team 2020^27^, https://www.r-project.org/) with the R survival, cmprsk, rms, and mstate packages for constructing the model as well as the nomogram, and the package pec for testing the predictive performance. A two-tailed *P*-value of < 0.05 was considered statistically significant in the current study.

### Ethics approval and consent to patients

Use of SEER is exempt from Institutional Review Board, and no informed consent is needed. The current study adhered to the 1964 Declaration of Helsinki and its amendments.

## Results

### Baseline characteristics

The baseline demographic and clinicopathologic characteristics of 5993 eligible patients are reported in Table 1. In brief, the median age at diagnosis was 62 years (interquartile range 54–69 years); the majority of patients were aged 50–74 years (4361, 72.8%), male (4477, 74.7%) and belonged to white race (3981, 66.4%). Most tumors were in T1 stage (4697, 78.4%) and without lymph node metastases (5849, 97.6%). Bone, liver and lung metastases were present in 15 (0.3%), 10 (0.2%) and 16 (0.3%) cases, respectively. With respect to the pathological grade, more cases were diagnosed as grade II (2586, 43.2%), followed by grade III (1640, 27.4%). The median follow-up time was 40 months (interquartile range 23–59 months). Up to the last follow-up date of December 31, 2015, a total of 706 (11.8%) patients had died, with 298 (5.0%) deceased from the cancer-related causes, and 408 (6.8%) from other causes.

**Table 1:** Baseline characteristics of patients with papillary renal cell carcinoma.

Variables	Total (N = 5993)	%
**Age at diagnosis (years)**		
Median (IQR)	62 (54–69)	
< 50	900	15.0
50–74	4361	72.8
≥ 75	732	12.2
**Sex**		
Male	4477	74.7
Female	1516	25.3
**Race**		
White	3981	66.4
Black	1792	29.9
Other races	220	3.7
**Tumor side**		
Left	2948	49.2
Right	3045	50.8
**Pathological grade**		
I	539	8.9
II	2586	43.2
III	1640	27.4
IV	150	2.5
Unknown	1078	18.0
**T stage**		
T1	4697	78.4
T2	692	11.5
T3	576	9.6
T4	28	0.5
**N stage**		
N0	5849	97.6
N1	144	2.4
**Bone metastases**		
No	5978	99.7
Yes	15	0.3
**Liver metastases**		
No	5983	99.8
Yes	10	0.2
**Lung metastases**		
No	5977	99.7
Yes	16	0.3

*IQR* interquartile range.

### Cumulative incidences of CSM and OCM

**Figure 2:**
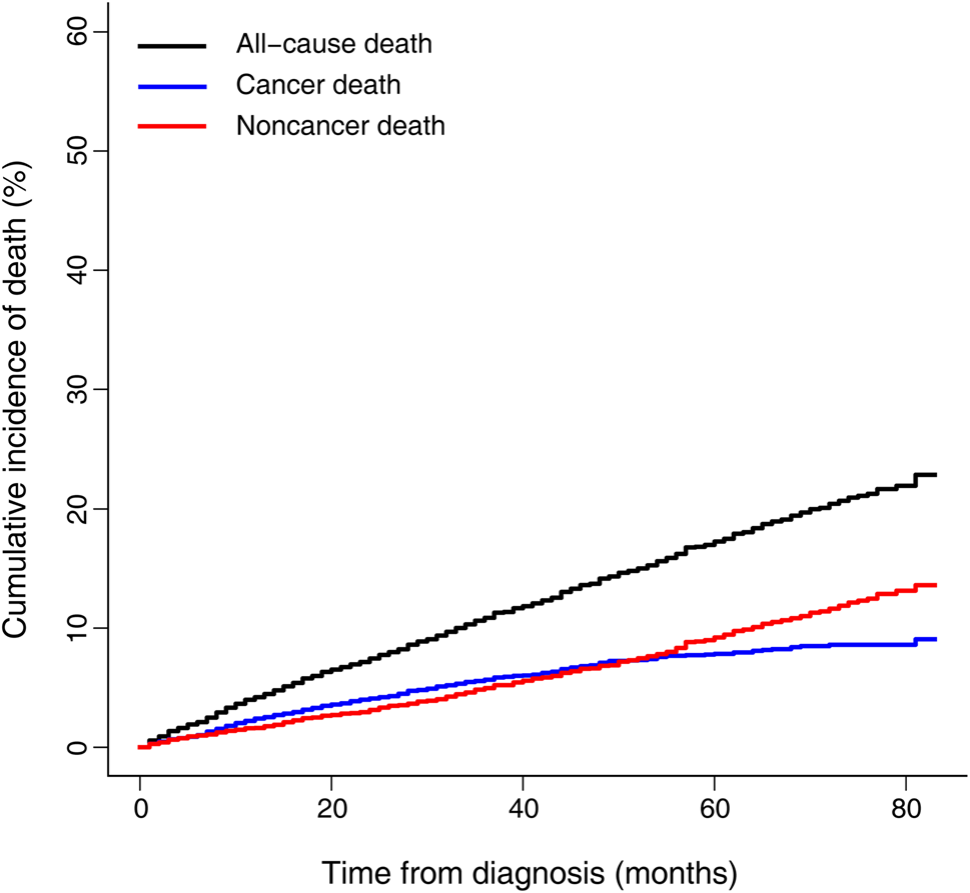
Cumulative incidences curves of cancer and noncancer death in the total cohort. The figure was performed using R software (version 4.0.3, R Core Team 2020, https://www.r-project.org/).

**Figure 3:**
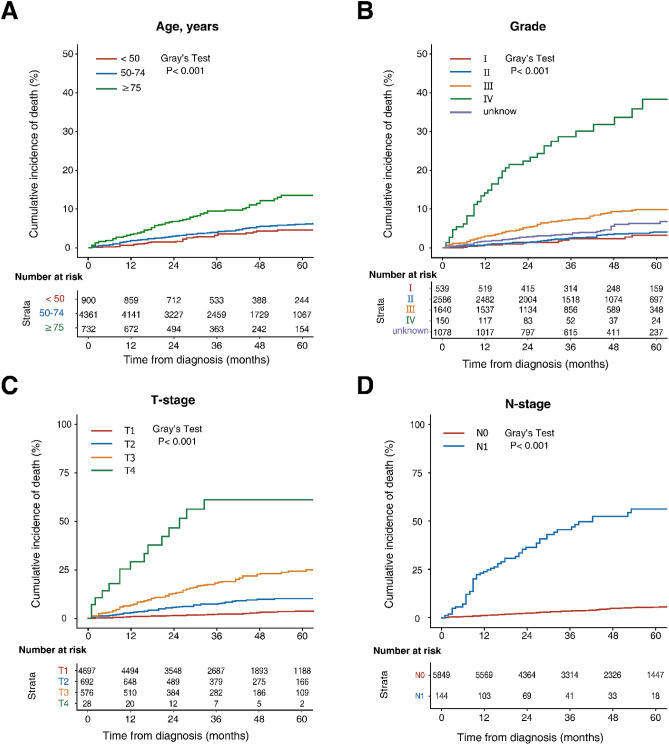
Cumulative incidences curves of CSM according to age (**A**), tumor grade (**B**), T stage (**C**) and N stage (**D**). The differences between groups were assessed by Gray’s test. *CSM* cancer-specific mortality. The figure was performed using R software (version 4.0.3, R Core Team 2020, https://www.r-project.org/).

**Figure 4:**
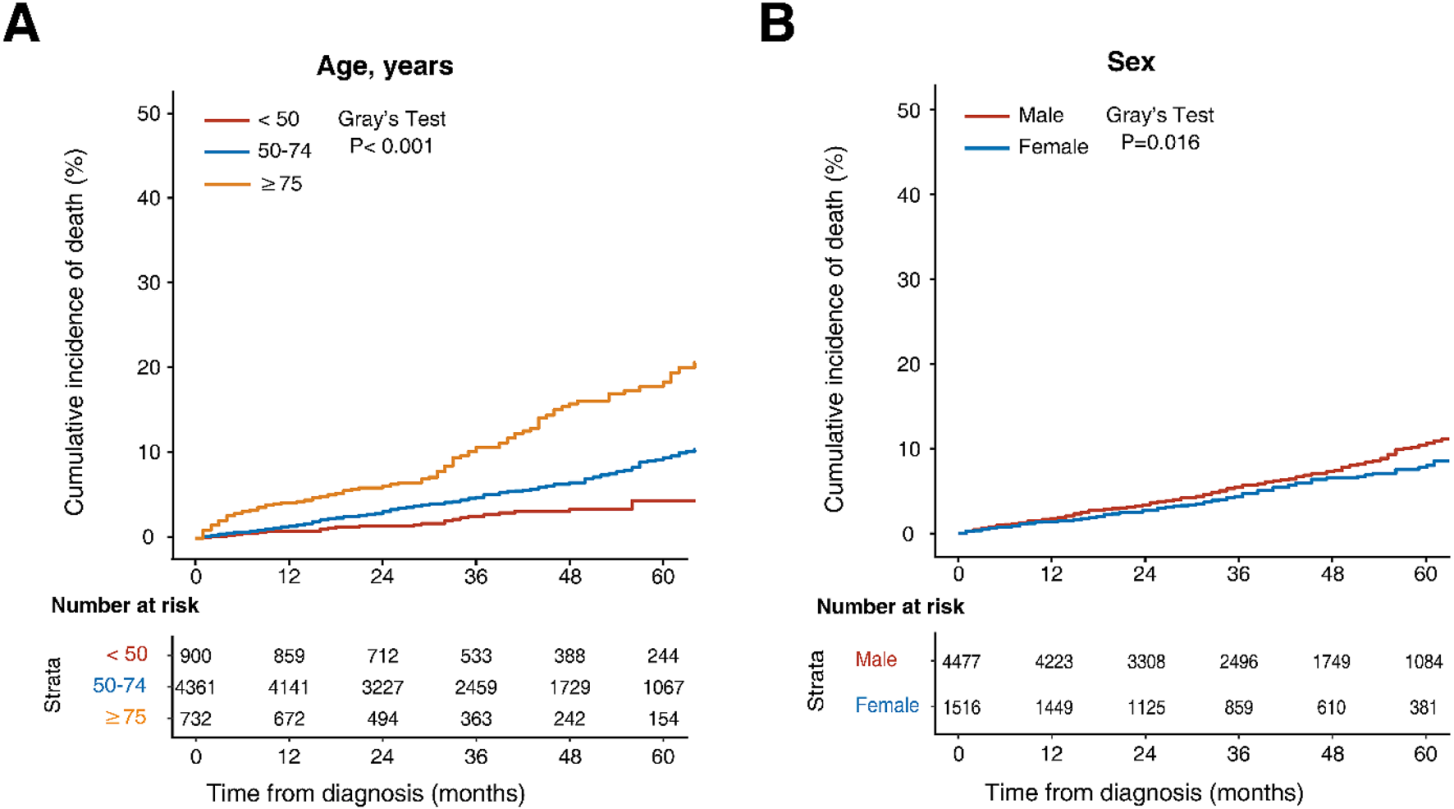
Cumulative incidences curves of OCM according to age (**A**) and sex (**B**). The differences between groups were assessed by Gray’s test. *OCM* other-cause mortality. The figure was performed using R software (version 4.0.3, R Core Team 2020, https://www.r-project.org/).

Overall, the 2-, 3- and 5-year estimated CSM rates were 3.2%, 4.4% and 6.5%, respectively; and the 2-, 3-, and 5-year OCM rates were 3.2%, 5.0% and 9.3%, respectively. The sum of the CSM and OCM rates was equal to the cumulative incidence of the all-cause mortality, and OCM progressively exceeded CSM during the whole follow-up period (Fig. 2). As shown in Fig. 3, the increased risks of CSM were significantly associated with older age, higher grade, higher T and N stage as assessed by Gray’s test (*p* < 0.001). Notably, the 2-, 3- and 5-year estimates of cumulative incidences were most pronounced among patients aged > 75 years for OCM (6.0%, 10.2% and 17.0%, respectively) (Fig. 4A). Males were more likely to die from other causes than females (*p* = 0.016, Fig. 4B). The CIF curves for CSM and OCM according to other variables were given in Supplementary Figs. S1 and S2, respectively.

### Univariable and multivariable competing risk analysis

On univariable analysis, age, race, tumor grade, T stage, N stage, and presence of bone, liver and lung metastases were significantly related to an increased risk of CSM (*p* < 0.05). Meanwhile, advancing age was a strong predictor of OCM (*p* < 0.001), both middle (sHR = 2.235, 95% CI 1.553–3.480) and elderly groups (sHR = 5.230, 95% CI 3.397–8.055) had higher OCM rates in comparison to the young group. Female gender (sHR = 0.744, 95% CI 0.585–0.948) showed lower competing risks of death than male gender (Supplementary Table S1). On multivariable analysis, age at diagnosis, tumor grade, T stage, N stage, and with or without bone metastases, liver metastases and lung metastases were retained as independent predictors of CSM following surgery (Table 2). Due to the number of significant variables observed in univariable analysis, a multivariable model was not built for OCM.

**Table 2:** Multivariable competing risk analysis for CSM.

Variables	Coefficient	SHR	95% CI	*p*
**Age at diagnosis (years)**				
50–74/< 50	0.443	1.557	1.057–2.292	0.025
≥ 75/< 50	1.092	2.980	1.921–4.623	< 0.001
**Race**				
Black/White	–	–	–	–
Others/White	–	–	–	–
**Pathological grade**				
II/I	0.128	1.137	0.626–2.065	0.670
III/I	0.827	2.287	1.281–4.084	0.005
IV/I	1.528	4.609	2.387–8.899	< 0.001
Unknown/I	0.586	1.798	0.970–3.333	0.063
**T stage**				
T2/T1	0.919	2.506	1.807–3.474	< 0.001
T3/T1	1.399	4.053	2.977–5.481	< 0.001
T4/T1	2.258	9.562	5.021–18.211	< 0.001
**N stage**				
N1/N0	1.476	4.375	3.045–6.287	< 0.001
**Bone metastases**				
Yes/no	1.723	5.600	2.351–13.336	< 0.001
**Liver metastases**				
Yes/no	0.945	2.573	1.173–5.646	0.018
**Lung metastases**				
Yes/no	1.849	6.351	2.848–14.164	< 0.001

*CSM* cancer-specific mortality, *CI* confidence interval, *sHR* subdistribution hazard ratio.

### Construction and validation of the nomogram

**Figure 5:**
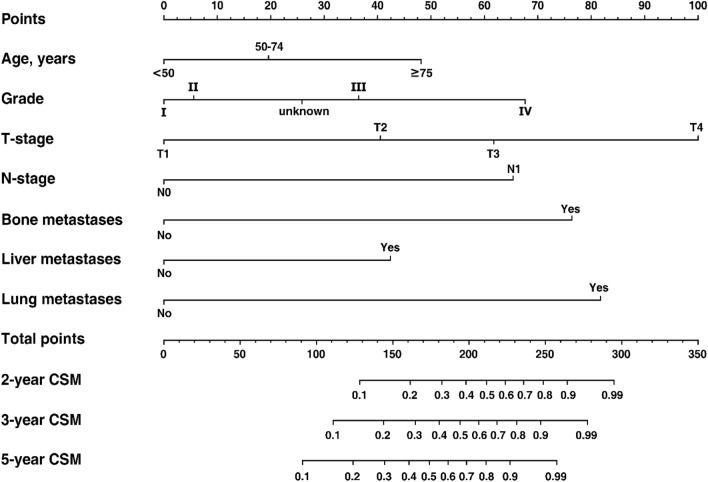
Competing risk nomogram predicting 2-, 3- and 5-year probabilities of CSM. *CSM* cancer-specific mortality. The figure was performed using R software (version 4.0.3, R Core Team 2020, https://www.r-project.org/).

**Figure 6:**
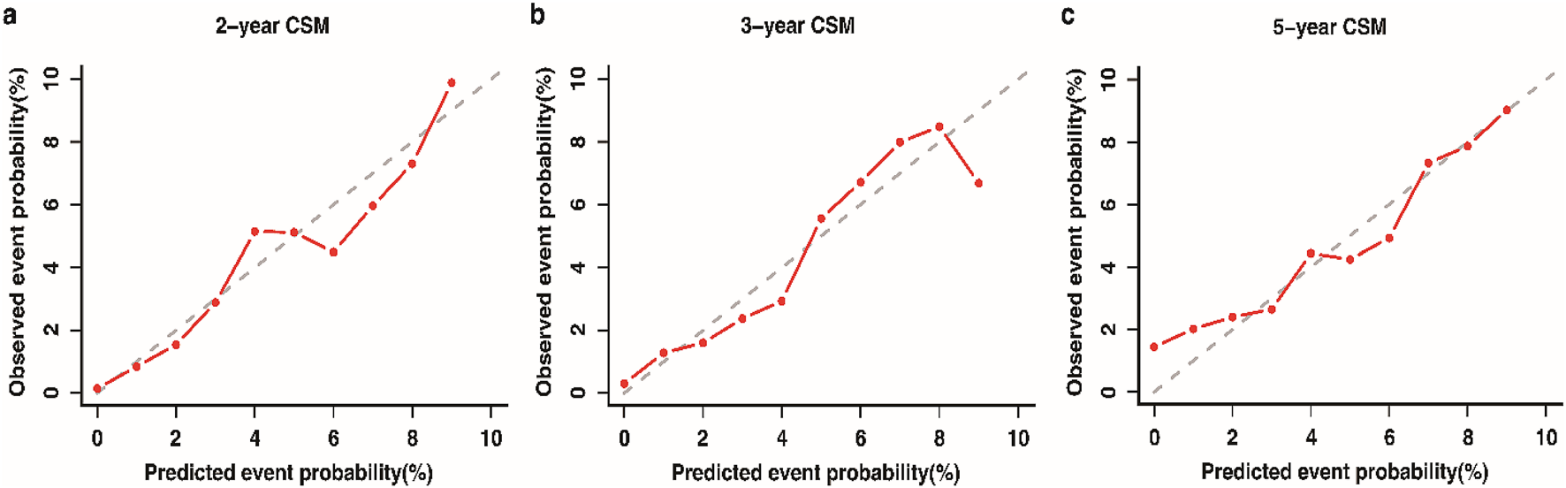
The calibration curves of the predicted probabilities and observed CSM rates. The 45-degree diagonal represents perfect agreements between the nomogram-predicted probabilities (X-axes) and the observed CSM rates (Y-axes). *CSM* cancer-specific mortality. The figure was performed using R software (version 4.0.3, R Core Team 2020, https://www.r-project.org/).

All the independent predictors were integrated to generate the nomogram predicting the probabilities of 2-, 3- and 5-years CSM (Fig. 5). Each variable was assigned to a value between 0 and 100 according to its contribution to the model. By summing up these values together, a total value could be obtained and then it was applied to predict the corresponding CSM rates through the probability scale. Using the bootstrap method for internal validation, our model demonstrated an AUC_t_ of 0.730 at 5-year for discrimination ability. The 2-, 3-, and 5-year calibration curves presented good agreements between the nomogram-predicted probabilities and the observed CSM rates, as the curves were close to the 45-degree diagonal (Fig. 6).

## Discussion

As mentioned previously, pRCC is the second most common subtype of RCC, which is a heterogeneous solid tumor consisting of type 1 and type 2^2^. Compared with ccRCC, the pathogenesis of pRCC is not associated with alterations in the *VHL* gene. Indeed, type 1 usually presents with mutations in the *MET* oncogene, whereas type 2 is primarily due to activation of the NRF2-ARE pathway^4^. Therefore, pRCC has distinct clinicopathological features and treatment response from ccRCC. Several groups have reported that pRCC exhibit a better prognosis than ccRCC using Kaplan–Meier methods, with lower TNM stage and tumor grade. Meanwhile, patients with pRCC are usually older than those with ccRCC at the time of diagnosis^10–12^. Notably, Keegan et al.^11^ retrospectively analyzed surgically treated RCC patients in the SEER database from 2000 to 2005, who observed a significant difference in CSS between pRCC and ccRCC subtypes, however there was little difference in OS. The authors speculated that this discrepancy may be related to the increased comorbidities due to the high incidence of end-stage renal disease in these population. In addition, it is unreasonable to treat patients experiencing competing events as the noninformative censoring in traditional statistical methods. When analyzing the data with competing risks, the overestimated risks provided by the Cox proportional hazards model may lead to overtreatment of patients. To date, there is still a lack of the competing risk analysis of pRCC patients following surgery. Our current study aimed to screen the independent predictors and construct a reliable competing risk nomogram to predict CSM of pRCC using a large-scale cohort from the SEER program.

Based on the univariable and multivariable competing risk regression models, age, tumor grade, T and N stage, and M status were identified as the independent predictors of CSM, in accordance with the previous well-accepted researches for RCC patients following surgery^5–7^. As shown in Fig. 5, T stage was the most significant independent predictor, followed by N stage and tumor grade, underscoring the prognostic significance of the 2010 AJCC staging system and the International Society of Urological Pathology consensus^28,29^. With respect to M status, we further analyzed the specific site of metastases, which was subdivided into lung, bone, and liver with reference to the method proposed by Hou et al.^30^, and all three covariates made significant contributions to the CSM, especially the occurrence of lung metastases. We thought that it could perform a more individualized prediction and surveillance of prognosis using this approach compared to others classifying patients as M0 or M1. In terms of the internal validation, our nomogram demonstrated good predictive performance with an AUC_t_ of 0.730 and excellent calibration curves.

Over the past decade, several prognostic models have been reported for pRCC patients after nephrectomy. In 2010, Klatte et al.^15^ established the first clinical nomogram for predicting postoperative disease-specific survival in pRCC patients, which included variables such as T stage, M stage, vascular invasion, tumor necrosis and initial symptom status. Although the predictive accuracies of internal and external validation were 93.6% and 94.2%, respectively, it is worth noting that these researchers only retrospectively analyzed a limited sample size from three institutions; thus, the findings may not be generally applicable. Therefore, it is not surprising that the C-index dropped to 0.72 in a recent external validation based on 1372 patients from a large multicenter pRCC database^31^. Moreover, using Cox methods without considering the impacts of competing events may have biased their results to some extent. Leibovich et al.^14^ published a risk stratification system assessing the disease progression and death from nonmetastatic pRCC treated with nephrectomy. Based on the status of nuclear grade, fat invasion and tumor thrombus, patients were categorized into three groups with low, intermediate, and high risk, respectively. Of note, dividing patients into different risk groups instead of providing a specific probability value might restrict the utility of the prognostic model in clinical practice. Recently, Klatte et al.^13^ reported another prognostic scoring system for nonmetastatic pRCC, namely the VENUSS (Venous extension, Nuclear grade, Size, T and N Stage) score, ranging from 0 to 11. This score was further externally validated with an independent cohort and it showed a 66.5% predictive accuracy at 5 years. One study reviewed a SEER cohort (N = 13,926) and developed a nomogram predicting the midterm to long-term prognosis in patients with pRCC, using the Cox regression method without differentiating the OCM^32^. In view of the similar data set, the variables they reported to predict OS were consistent with our results. Additionally, the authors revealed that most patients were > 60 years old and had higher all-cause mortality rates than younger patients.

We also found that old age was a significant predictor of OCM, which was consistent with previous studies evaluating the impact of age on the clinical course and prognosis in elderly patients. Specifically, one study reviewed the patients with localized and surgically treated RCC in SEER, and it indicated that age was strongly related to noncancer death^17^. Likewise, Borgmann et al. reported the survival outcomes of 2189 pRCC patients collected from an international multi-institution database. Based on the competing risk analysis, the authors demonstrated that older age and poor Eastern Cooperative Oncology Group (ECOG) performance status were significantly predictive of OCM^20^. In our cohort, the incidence of noncancer death was quite significant and most prominent among patients aged > 75 years. In fact, kidney cancer is most prevalent between the ages of 60 and 70 years^33^, and these patients are more prone to chronic comorbidities and have a poor nutritional condition. Irrespective of the differences in biological behavior and clinicopathologic features of pRCC across age groups, elderly patients may not be able to tolerate invasive surgery and may die from noncancer death before benefiting from the treatment. On the other hand, as a result of the potentially longer clinical course and life expectancy compared to conventional ccRCC^19^, pRCC patients may frequently experience age-related competing events. Due to these reasons, clinicians should be aware of the probable competing risks of death and make trade-offs between risks and benefits before treatment, especially while managing older patients with nonmetastatic pRCC.

In the study, we analyzed the survival data of pRCC patients using a large population-based cohort, and provided a visual tool for clinicians and patients to quantitatively assess the CSM, which showed an impressive performance and could be used in both the preoperative setting and postoperative follow-up. Critically, we evaluated and differentiated the effects of variables on each type of event over time based on the approach of competing risk regression, which has been increasingly recommended for constructing prognostic models of survival data^16^. In addition, all seven variables included in our nomogram were easily accessible in diagnosis, and thus our predictive model was expected to be widely applied in busy clinical practice. However, several limitations of the current study should be pointed out. Firstly, the specific histological subtypes of pRCC (type 1 and type 2) were not detailed in the SEER database and could not be incorporated into the analyses. It was thought that the type 2 pRCC usually had higher TNM stage and tumor grade, exhibiting a worse prognosis compared with type 1^28^. Nonetheless, several studies suggested that histological subtypes did not affect the outcomes after adjusting for potential confounders, either in metastatic or non-metastatic pRCC patients^34–37^. Secondly, we failed to further investigate the independent factors and to develop the predictive nomogram for OCM since other relevant variables, such as laboratory indices, clinical symptoms, comorbidities, and performance status, were not documented in the database. Also, as the limited information provided by the SEER database, we could only exclude cases with bilateral tumors and were unable to identify the multiple lesions. However, several studies indicated that the multiple lesions were not significantly associated with the tumor stage and grade, nor with the OS in pRCC patients after surgery^38–40^. Thirdly, due to the retrospective nature of our study, the histopathological data could not be centrally reviewed, and some subtypes of RCC with similar papillary or pseudopapillary structures may have been misclassified as type 2, which may have resulted in some degree of bias^28^. Finally, although our model showed excellent predictive performance in internal validation, an independent cohort from other centers is required to further validate our findings in the future.

## Conclusions

This study performed a competing risk analysis in pRCC patients following surgery based on the SEER database. Age, tumor grade, T and N stage, and with or without bone, liver and lung metastases were identified as independent predictors for CSM. Besides, old age was associated with a high risk of OCM and it especially impacted the long-term benefits of treatment for elderly patients. To the best of our knowledge, we constructed the first competing risk nomogram to calculate the probability of 2-, 3- and 5-year CSM, which can provide a reference for decision-making, patient counselling and screening appropriate subjects for adjuvant trials.

## Supplementary Information

Supplementary Information.

## Data Availability

The SEER database was available from: www.seer.cancer.gov.
