# Supplementary material for: The first competing risk survival nomogram in patients with papillary renal cell carcinoma
Source: Sci Rep. 2021 Jun 4;11:11835. doi: 10.1038/s41598-021-91217-z (PMC8178392; doi:10.1038/s41598-021-91217-z)
Supplement: Supplementary file 1 — Supplementary Information. [file 41598_2021_91217_MOESM1_ESM.pdf]

The first competing risk survival nomogram in patients with papillary renal cell carcinoma

Xing Su[1]*, Niu-Niu Hou[2]*, Li-Jun Yang[1]*, Peng-Xiao Li[3]*, Xiao-Jian Yang[1], Guang-Dong Hou[1], Xue-Lin Gao[1], Shuai-Jun Ma[1], Fan Guo[1], Rui Zhang[1], Wu-He Zhang[4], Wei-Jun Qin[1#] ＆ Fu-Li Wang[1#]

[1] Department of Urology, Xijing Hospital, Fourth Military Medical University, Xi'an 710032, China

[2] Department of Thyroid, Breast and Vascular Surgery, Xijing Hospital, Fourth Military Medical University, Xi'an 710032, China

[3] Department of Cardiology, Xijing Hospital, Fourth Military Medical University, Xi'an 710032, China

[4] Department of Urology, the 986th Hospital of Air Force, Xi'an 710054, China

* Xing Su, Niu-Niu Hou, Li-Jun Yang and Peng-Xiao Li contributed equally to this work.

[#] Correspondence and requests for materials should be addressed to Fu-Li Wang (email: wangfuli98@163.com) or Wei-Jun Qin (email: qinwj@fmmu.edu.cn)

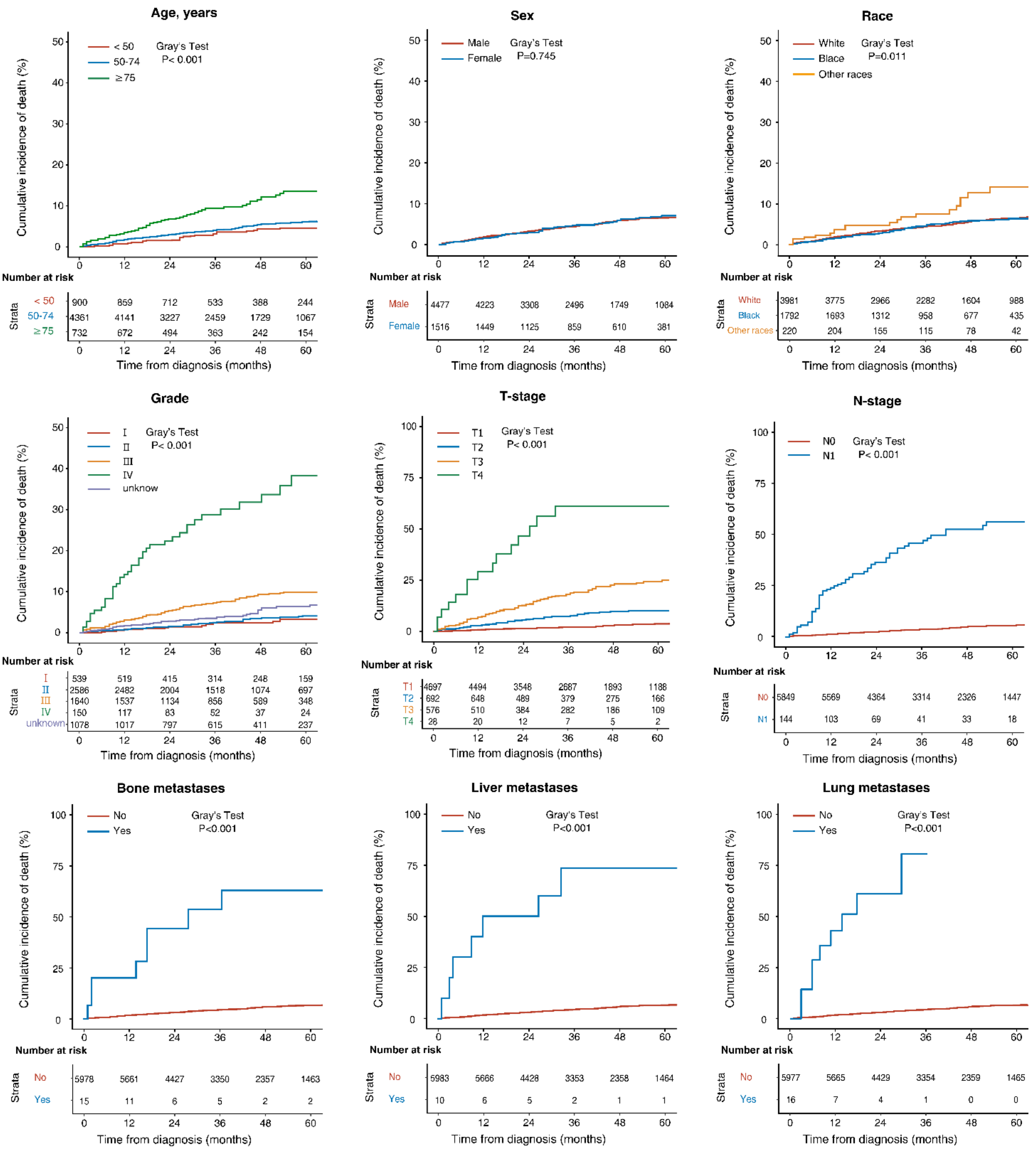

Supplementary Figure S1. Cumulative incidences curves of CSM according to baseline demographic and clinicopathologic characteristics. The differences between groups were assessed by Gray's test. *CSM* = cancer-specific mortality. The figure was performed using R software (version 4.0.3, R Core Team 2020, https://www.r-project.org/).

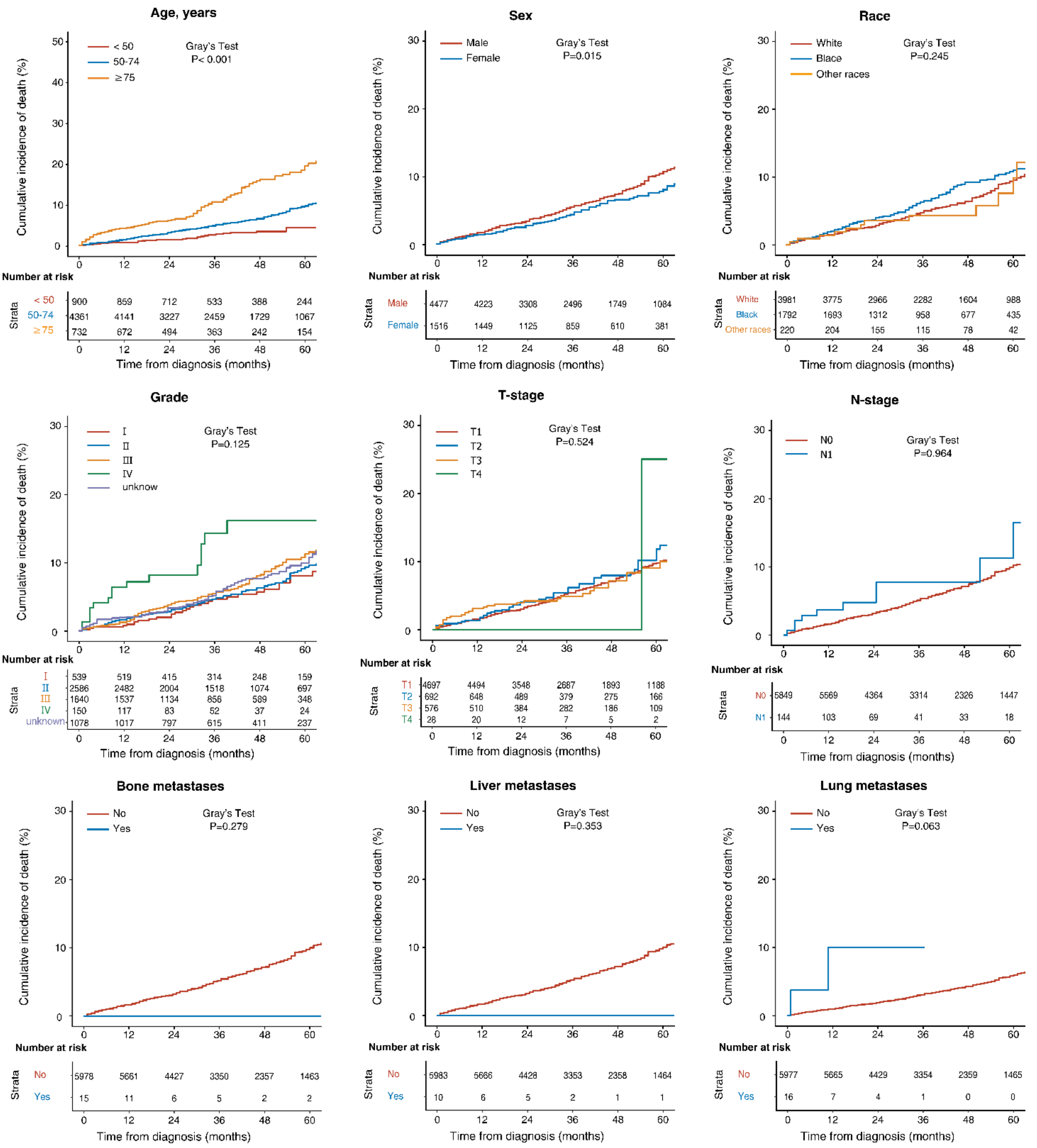

Supplementary Figure S2. Cumulative incidences curves of OCM according to baseline demographic and clinicopathologic characteristics. The differences between groups were assessed by Gray's test. *OCM* = other-cause mortality. The figure was performed using R software (version 4.0.3, R Core Team 2020, https://www.r-project.org/).

Variables	CSM			OCM		
	SHR	95% CI	*p*	SHR	95% CI	*p*
Age at diagnosis, years						
50-74/<50	1.390	0.974-2.038	0.092	2.235	1.553-3.480	<0.001
≥75/<50	3.029	1.978-4.640	<0.001	5.230	3.397-8.055	<0.001
Sex						
Female/ Male	1.044	0.807-1.351	0.740	0.744	0.585-0.948	0.017
Race						
Black/White	0.958	0.741-1.239	0.740	1.189	0.965-1.464	0.100
Others/White	1.961	1.241-3.098	0.004	0.950	0.546-1.654	0.860
Tumor side						
Right/Left	1.013	0.807-1.271	0.910	0.969	0.798-1.176	0.750
Pathological grade						
Ⅱ/Ⅰ	1.243	0.675-2.290	0.480	1.224	0.831-1.803	0.310
Ⅲ /Ⅰ	3.732	2.066-6.740	<0.001	1.365	0.913-2.039	0.130
Ⅳ/Ⅰ	15.468	8.117-29.477	<0.001	2.156	1.161-4.005	0.015
Unknow/Ⅰ	2.086	1.109-3.923	0.023	1.318	0.862-2.015	0.200
T stage						
T2/T1	3.139	2.282-4.318	<0.001	1.132	0.849-1.508	0.400
T3/T1	7.889	6.085-10.228	<0.001	0.845	0.589-1.214	0.360
T4/T1	33.220	18.781-58.760	<0.001	0.511	0.076-3.443	0.490
N stage						
N1/N0	15.511	11.722-20.525	<0.001	1.038	0.550-1.958	0.910
bone metastases						
Yes/No	17.511	8.395-36.529	<0.001	-	-	-
liver metastases						
Yes/No	24.117	10.446-55.682	<0.001	-	-	-
lung metastases						
Yes/No	23.790	11.886-47.617	<0.001	2.367	0.547-10.252	0.250

Supplementary Table S1. Univariable competing risk analysis of baseline demographic and clinicopathologic characteristics for CSM and OCM. *CSM* = cancer-specific mortality, *OCM* = other-cause mortality, *CI* = confidence interval, *sHR* = subdistribution hazard ratio.
